# Panuveitis-like reaction following encircling laser retinopexy/cerclage in a 21-year-old male

**DOI:** 10.1186/s12348-017-0139-y

**Published:** 2017-10-16

**Authors:** David Francis Fullon Chan, Milagros Herrera-Arroyo, Darby E. Santiago, Teresita R. Castillo, Ma Florentina Q. Fajardo-Gomez

**Affiliations:** Department of Ophthalmology and Visual Sciences, University of the Philippines Manila-Philippine General Hospital, 5th Floor, Sentro Oftalmologico Jose Rizal, Taft Avenue between Padre Faura and Pedro Gil Streets, Manila, Philippines

**Keywords:** Encircling laser retinopexy, Laser cerclage, Retinal detachment prophylaxis, Retinal detachment, Retinal laser/photocoagulation, Post-laser inflammation

## Abstract

**Background:**

Severe vision-impairing ocular inflammation is rarely reported following extensive laser. Previous cases have involved retinal photocoagulation for diabetic retinopathy resolving over days. This report documents a rare instance of this where encircling retinopexy/cerclage was done as fellow eye retinal detachment prophylaxis in a patient with no overt comorbidities.

**Results:**

A panuveitis-like reaction with severe, near-total visual impairment was documented 1 day following single-sitting encircling laser retinopexy/cerclage done as fellow eye prophylaxis for a 21-year-old male presenting with total retinal detachment in the other eye. Pre-laser findings were unremarkable, other than an equatorial ring of fine vitreous condensations. Pre-laser vision of 20/20 uncorrected decreased to hand motion, light perception on all quadrants, accompanied by severe anterior segment inflammation with hypopyon, retrolental membranes, vitreous cells, and choroidal effusion/suspicious exudative retinal detachment on B-scan ultrasound and ultrasound biomicroscopy. Combination of oral, topical, and depot steroids resulted in restoration of vision by 1 month post-laser, but with persistent anterior segment inflammation and retrolental membranes at month 2 post-laser.

**Conclusions:**

The atypically inordinate degree of post-laser inflammation and multiple sequelae following encircling retinopexy/cerclage as retinal detachment prophylaxis, in this case, demonstrate the potency and risks of retinal photocoagulation. The value of pre-laser assessment for potential risk factors, caution and mindfulness in conducting the intervention, and, the value of prudent and thorough follow-up are exhibited in this case.

## Findings

### Introduction

Side-effects and complications of retinal photocoagulation are well documented in the literature. Among its manifestations is uveitis, with cases of hypopyon uveitis following panretinal photocoagulation for diabetic retinopathy reported sporadically. Extensive laser is known to result in rupture of tissue, hemorrhage, and inflammatory reactions such as choroidal effusions [1–4]. We report a severe, panuveitis-like reaction to single-sitting encircling laser retinopexy/cerclage as prophylaxis for retinal detachment in a young male with no established comorbidities and only equatorial vitreous condensations as an abnormal finding in the treated eye prior to laser.

### Case report

A 21-year-old male with no established systemic illnesses was referred to our service for a 1-year history of blurring of vision in the right eye. The blurring was curtain-like, occurring 4 days after he was knocked down in a landslide, prone, with no loss of consciousness. It progressed over the past year with no interventions. Best-corrected visual acuity was hand motion, light perception in all quadrants in that eye. The anterior segment was unremarkable having formed chambers, no iris changes nor keratic precipitates. The lens was clear and stable. A high, mobile retinal detachment with no cells nor flare in both anterior and posterior segments was observed.

The left eye meanwhile had 20/20 uncorrected visual acuity, with a similarly unremarkable anterior segment and lens. The fundus of the left eye was found to have an unremarkable posterior pole, having clear media, a cup-disc ratio of 0.3, arterio-venous ratio of 2:3, normal course of vessels with no perivascular changes, a normal-looking foveal reflex, and without intraretinal hemorrhages or exudates. A thin ring of feathery, gray-white vitreous material, signed out as benign vitreous condensations/vitreoretinal interface changes, was seen anterior to the equator. Intraocular pressures (IOP) in mmHg were 12 for the right and 13 for the left.

Scleral buckling and vitrectomy were offered for the right eye, and encircling laser retinopexy was offered for the left eye. The patient decided against surgery for the right eye and agreed to laser treatment for the left eye.

Encircling laser retinopexy was done on the left eye using an Iridex OcuLight TX green 532-nm laser, delivered via a Mainster PRP 165 contact lens with spot size of 200 um, power of 130 mW, duration of 180 ms, and interval of 160 ms. A honeycomb-confluent pattern 3–4 burn widths was created on and around the area of condensations with 1352 shots/spots. Polymyxin + neomycin + dexamethasone 10,000 u/5.5 mg/1 mg/mL eye drops every 2 hours was then advised.

**Fig. 1:**
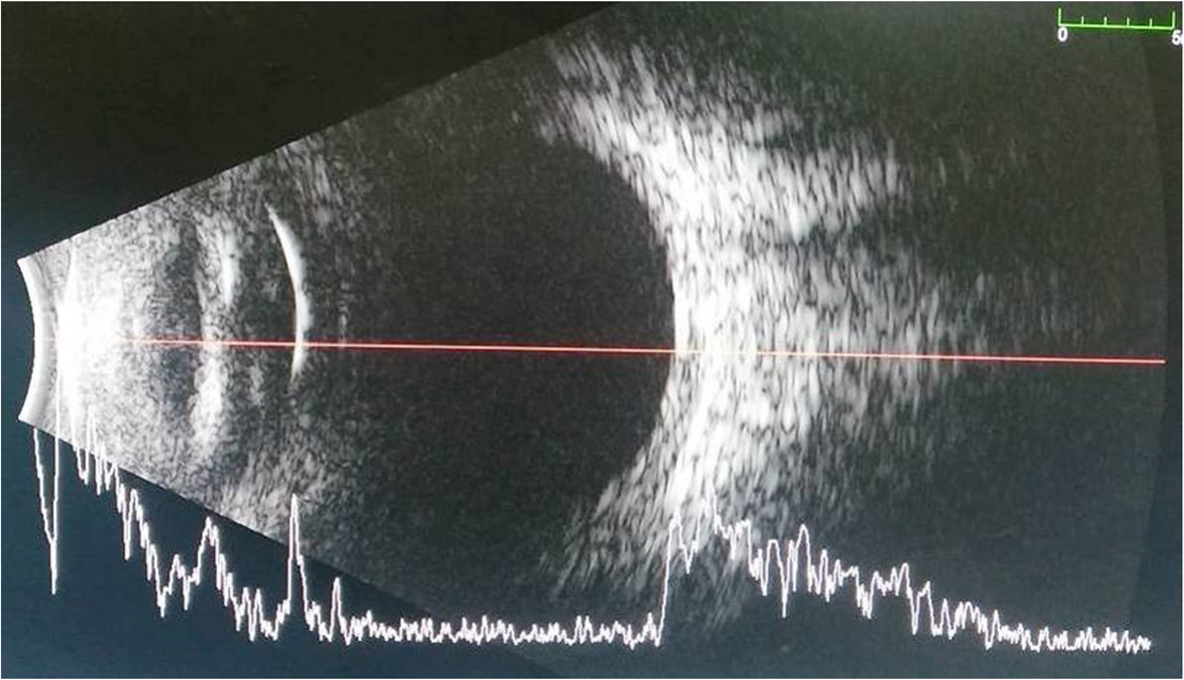
Day 2 post-laser B-scan ultrasound, axial horizontal, documents low amplitude vitreous echoes with good aftermovement and a prominent choroid

On follow-up, 1 day later, the patient reported acute vision loss accompanied by redness, pain, and tearing in that eye a few hours following laser. Best-corrected visual acuity on consult was documented as hand motion, light perception in all quadrants. An anterior segment reaction of + 3 brown cells and + 2 flare was observed, with dense vitreous haze on dilated indirect ophthalmoscopy totally prohibiting appreciation of fundus details. IOP was 20 mmHg, compared to 10 mmHg in the other eye. Topical medications were shifted to prednisolone acetate 10 mg/mL every 2 hours, atropine sulfate 1% three times a day, and oral acetazolamide 125 mg two times a day. B-scan ultrasound on day 2 post-laser demonstrated low amplitude and mobile echoes in the mid to posterior vitreous, as well as a prominent choroid (Fig. 1). Treatment was maintained, on the working impression of a severe, acute post-retinal photocoagulation uveitis probably with vitreous hemorrhage.

**Fig. 2:**
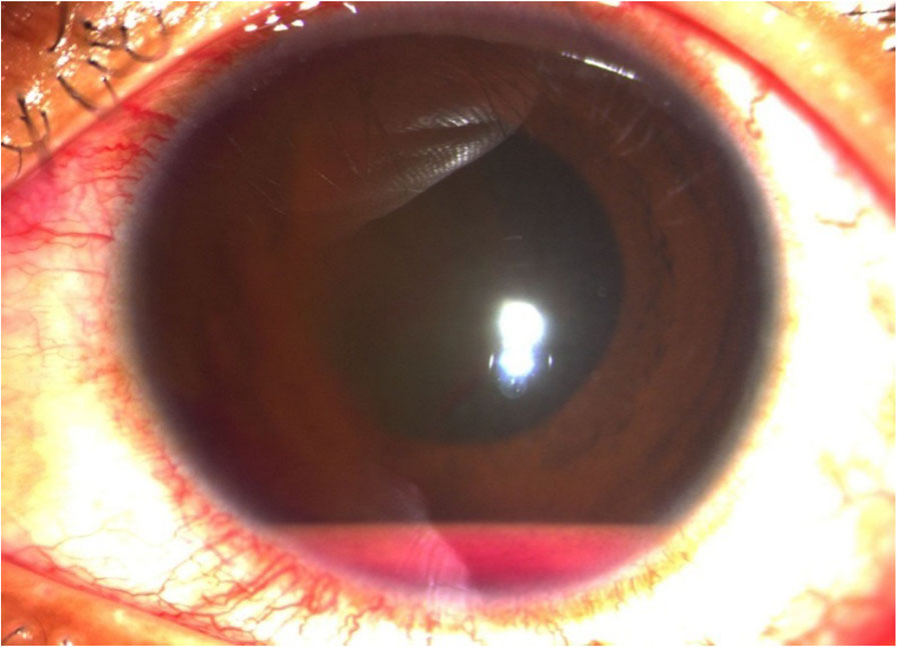
Day 4 post-laser, documents a 1.0mm blood-tinged hypopyon with accompanying best-corrrected visual acuity (BCVA) of hand motion, light perception in all quadrants

**Fig. 3:**
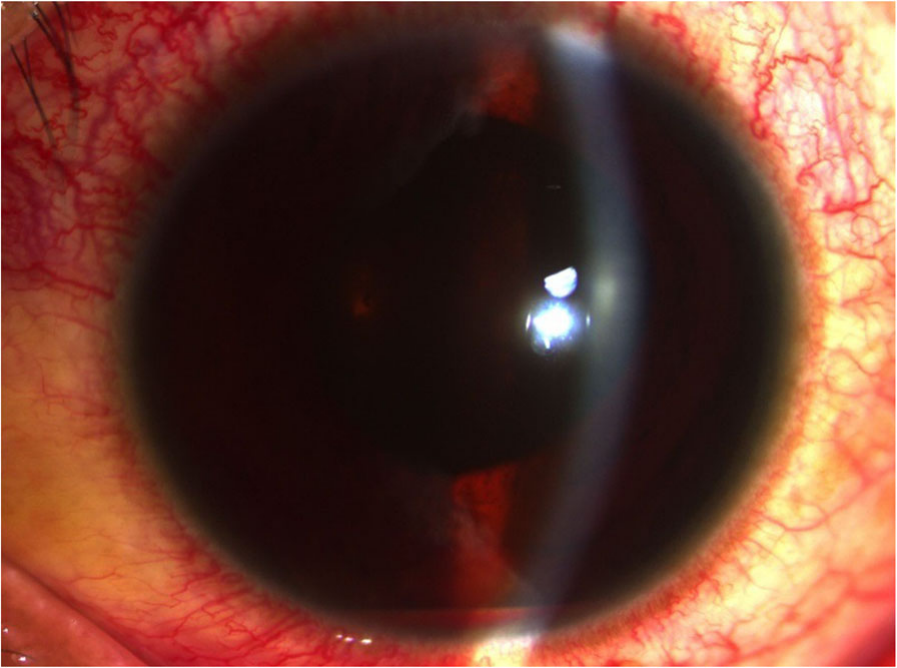
On Day 8 post-laser, four days following subconjunctival injection of triamcinolone acetonide, 10mg, hypopyon is seen reduced, with a marginally decreased anterior chamber cell and flare

The patient returned again on day 4 post-laser, demonstrating a 1-mm blood-tinged hypopyon and IOP of 10 mmHg (Fig. 2). Topical prednisolone acetate was increased to one drop hourly, and topical moxifloxacin 5 mg/mL every 3 hours was started. Subconjunctival triamcinolone acetonide (10 mg) was injected in the superior conjunctiva. Oral prednisone 60 mg daily was started on day 8 post-laser, following work-up revealing asymptomatic bacteriuria, elevated serum white blood cell counts with neutrophilic predominance, and unremarkable chest and lumbosacral radiographs. Gradual decrease of anterior chamber reaction then followed, with cells and flare going down to between + 1 and + 2, and the hypopyon resolving over the following 5 days (Fig. 3).

**Fig. 4:**
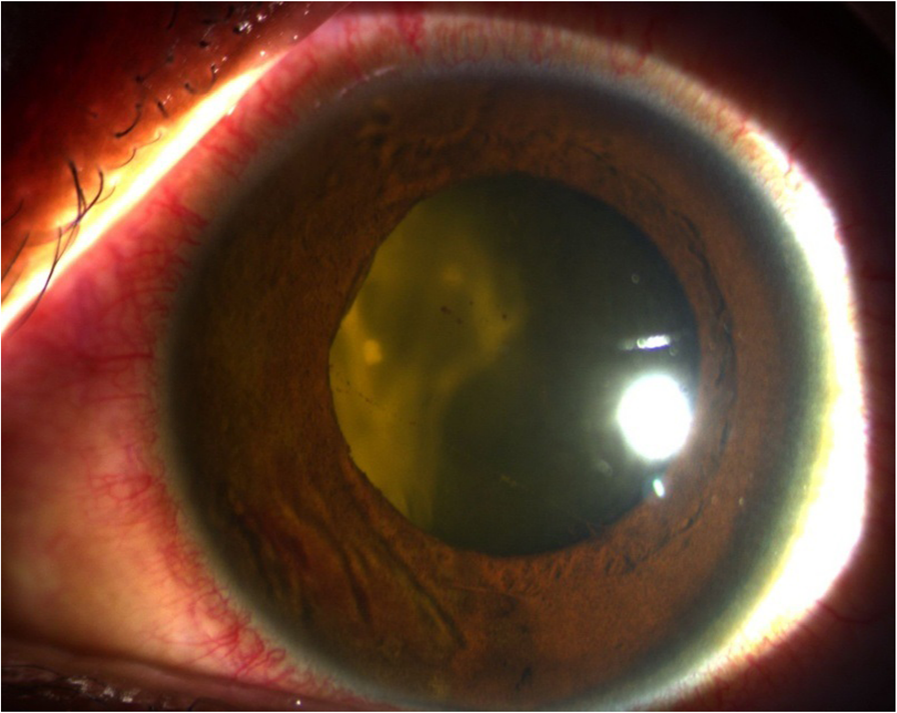
On day 15 post-laser, seen are beginning nasal papillary synechiae, retrolental membranes, decreased anterior chamber reaction, homogeneous gray haze on indirect ophthalmoscopy, accompanied by BCVA of hand motion, light perception in all quadrants

**Fig. 5:**
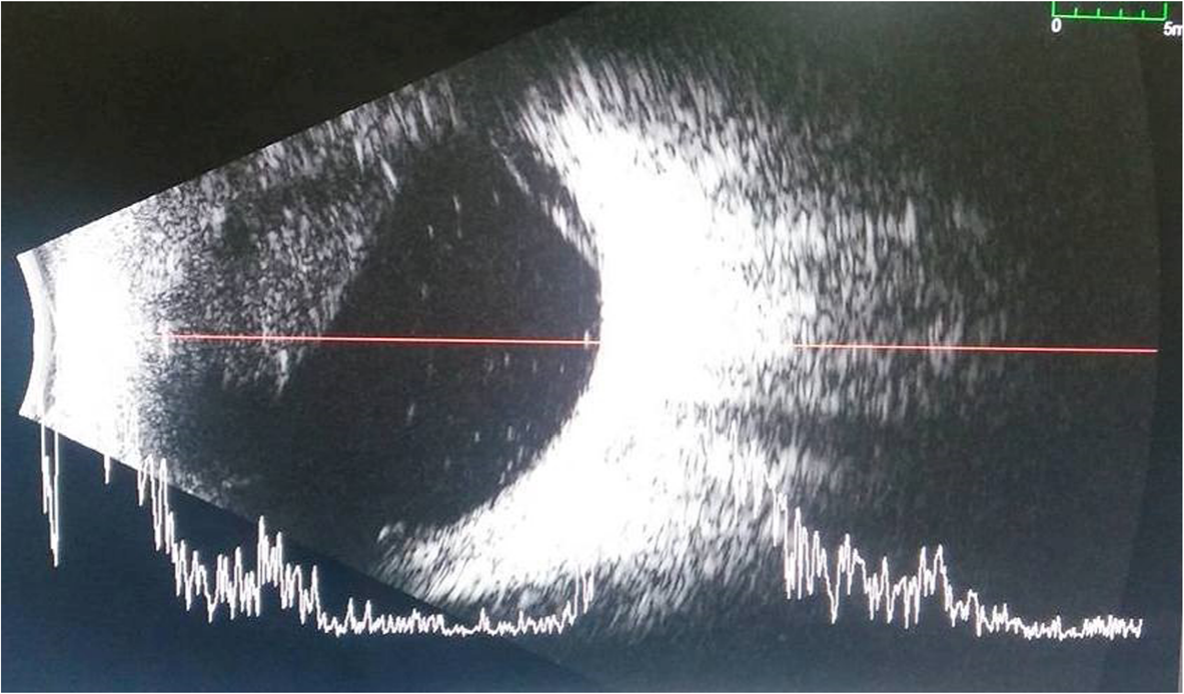
Day 15 post-laser B-scan ultrasound, axial horizontal documents dense, mobile retrolental/anterior vitreous echoes, mobile dot echoes with increasing amplitude in the mid-posterior vitreous, and a band echo suspicious for exudative retinal detachment on the nasal wall

**Fig. 6:**
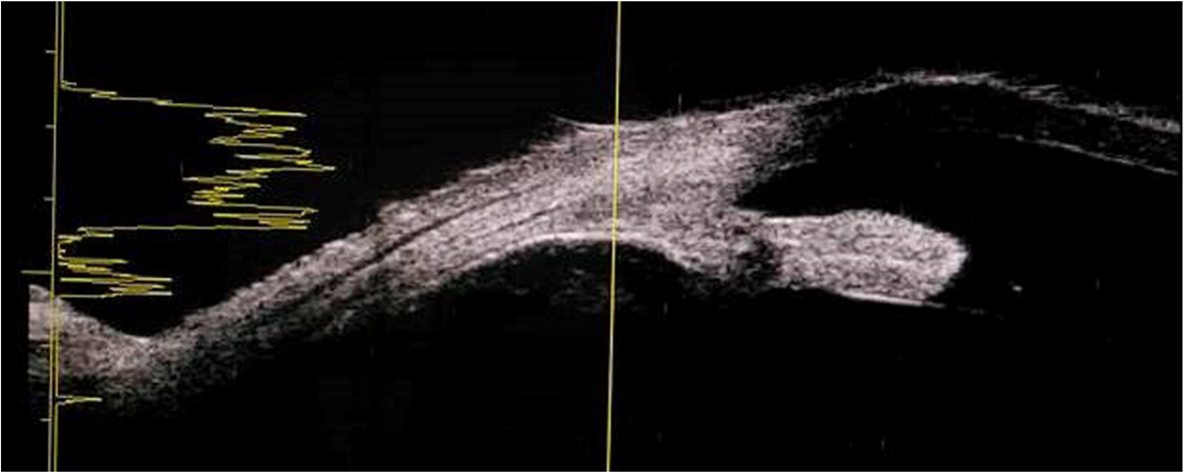
Day 15 post-laser UBM at ~8 o’clock documents ciliary body swelling, cystic morphology, and echoes occupying the space between the iris and lens

On week 3 (day 15) post-laser, however, evidence of pupillary synechiae and apparent swelling of the iris were noted from the 6 to 12 o’clock areas. Dense, mobile, gray-brown retrolental sheets/membranes were also observed (Fig. 4). B-scan ultrasound demonstrated dense, low-moderate amplitude mobile clumps in the anterior vitreous and more prominent dot echoes in the mid to posterior vitreous, as well as mobile mid-amplitude echoes in the nasal wall, suspicious for exudative retinal detachment (Fig. 5). Ultrasound biomicroscopy demonstrated a ciliary body cyst-like lesion and swelling, as well as echoes in the space between the iris and lens from 4 to 10 o’clock (Fig. 6). Oral immunosuppressive therapy owing to the severity of inflammation and pars plana vitrectomy to clear the visual axis were contemplated at this point, per consultations with ten consultants from our Vitreoretina and Uveitis services. Physicians in charge opted to forego additional medication pending further developments and defer surgical intervention for fear of pushing the eye into phthisis.

**Fig. 7:**
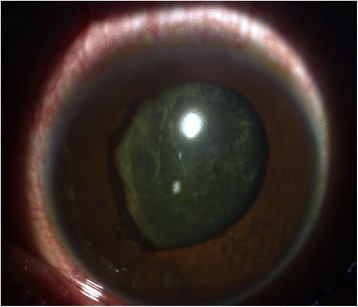
Day 36 post-laser. Uncorrected VA 20/70, best corrected to 20/20

On week 4 post-laser, B-scan ultrasound demonstrated the same gross features but decreased amplitude of echoes. Fundus details were first seen on indirect ophthalmoscopy at day 30 post-laser, with no gross differences from baseline appreciated save for anteriorly projecting gray-white sheets at the mid-periphery/roughly the area of laser work, prohibiting view of more anterior/peripheral regions. 20/200 vision with and without pinhole was documented on day 27 post-laser. 20/70 vision correcting to 20/20 on pinhole was first documented on day 36 post-laser (day 28 post-oral steroids) (Fig. 7). Oral prednisone was gradually adjusted to 40–50 mg/day by week 6.

20/20 uncorrected vision was first documented on week 8 post-laser (day 59), with persistence of anterior chamber + 1–2 cells and flare and the retrolental membrane. The iris had become more velvety, with more pronounced pupillary synechia. Gonioscopy revealed flat iris plane and a deep chamber that opens on dynamics on all quadrants. Anterior subcapsular cataract was noted along the margins of the synechiae (Fig. 11).

**Fig. 8:**
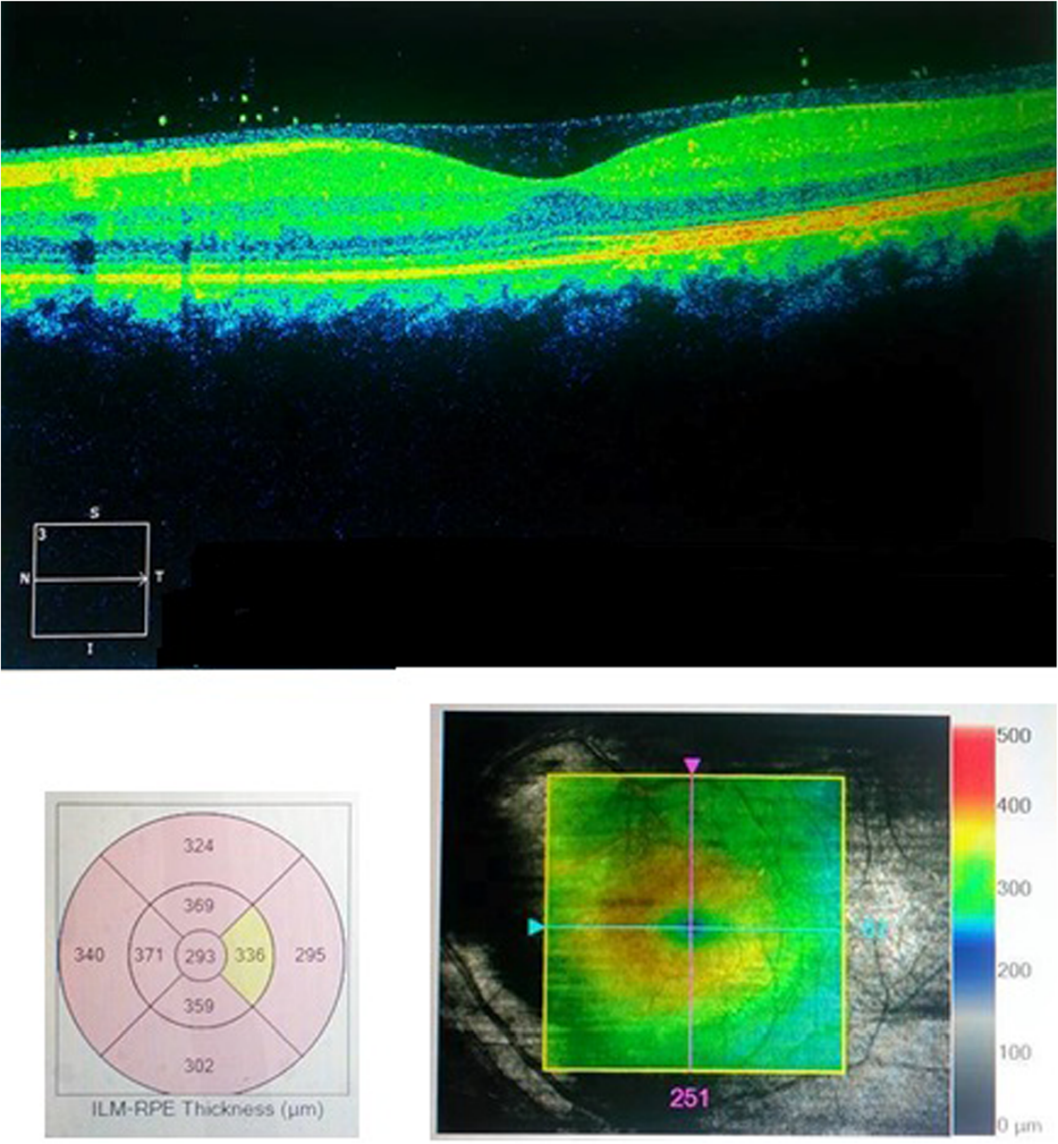
Day 56 Macular OCT demonstrates generalized macular thickening, preservation of the foveal contour, and prominent posterior hyaloid face detached over the foveal depression

**Fig. 9:**
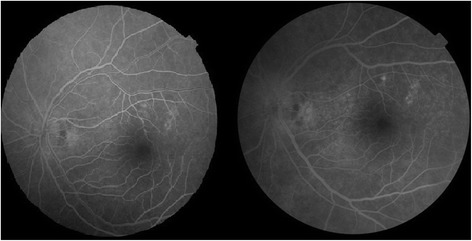
Day 56 Fluorescein angiogram, early venous (left) and recirculation (right) phases, demonstrates non-specific parafoveal staining superiorly, with no overt delays, evidence of vasculitis, macular edema nor ischemia

**Fig. 10:**
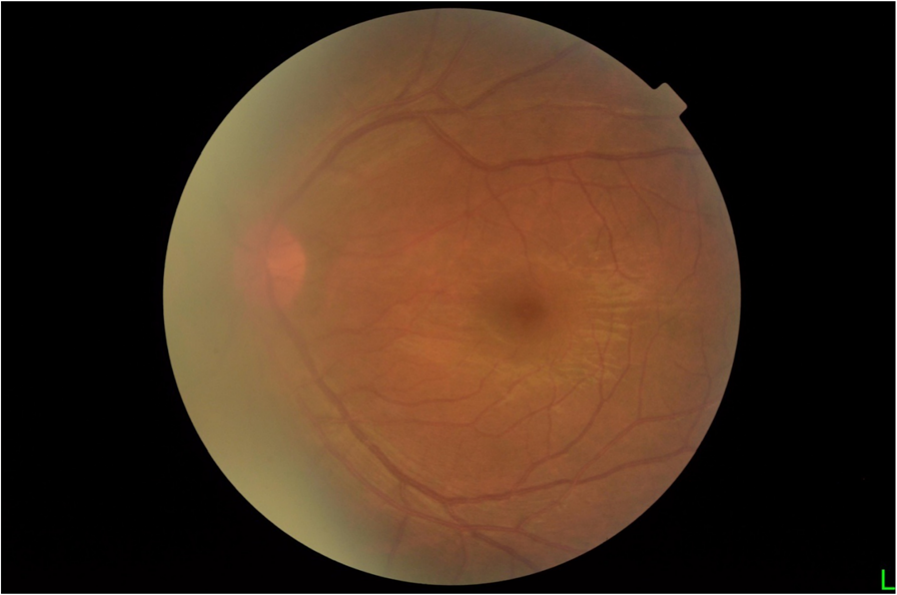
Day 56 fundus photo

Macular OCT using a Zeiss Cirrus HD-OCT 5000 demonstrated generalized macular thickening greater than the 99th percentile, with preserved foveal anatomy and a very visible posterior hyaloid face, detached above the foveal depression (Fig. 8). Fluorescein angiography demonstrated normal dye transit with no evidence of vasculitis, phlebitis, delayed filling, gross macular edema nor ischemia; only focal non-specific parafoveal staining was noted (Figs. 9 and 10). B-scan ultrasound demonstrated near-total resolution of the previously observed lesions, with the mid to posterior vitreous dot echoes being most prominent.

**Fig. 11:**
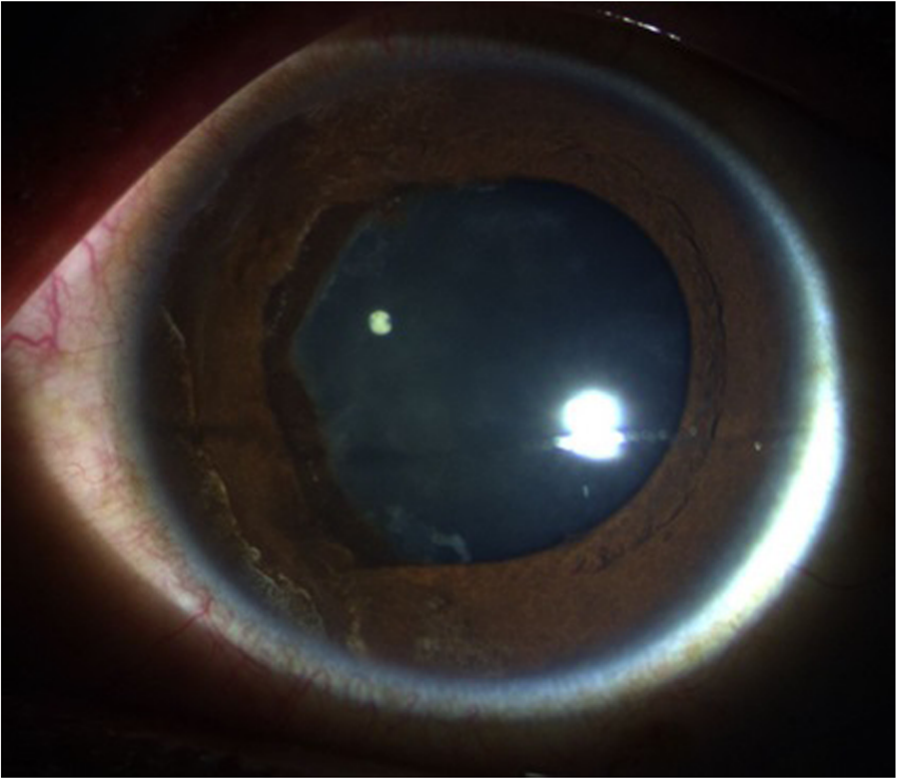
Day 75 post-laser anterior segment slit lamp photograph, demonstrating velvety iris, pronounced pupillary synechia with anterior subcapsular cataract along its margins, persistence of anterior chamber + 1–2 cells and flare, and retrolental membranes with accompanying uncorrected visual acuity of 20/20

**Fig. 12:**
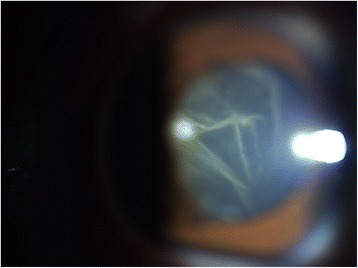
Day 75 post-laser anterior segment slit lamp photograph demonstrating flapping retrolental membranes viewed while on downgaze, with accompanying uncorrected visual acuity of 20/20

Eleven weeks post-laser, still on the aforementioned medications, IOP rose to the low thirties for the eye with retinal detachment and mid-twenties for the treated eye. Glaucoma therapy, including laser iridotomy due to creeping papillary synechiae, was contemplated at this point. Two glaucoma service consultants examined the patient and ordered timolol maleate 5 mg/mL twice a day to be started. Later, phacoemulsification instead of iridotomy was suggested, pending resolution of inflammation. IOP decreased in the following 2 days to single digits on the eye with detachment and high teens on the treated eye (Figs. 11 and 12).

Over the 1-month course, systemic work-up included the following, all returning within normal limits: sputum microscopy for acid-fast bacilli, tuberculin skin-testing, serum fasting blood sugar, hemoglobin, Hepatitis B sAg (nonreactive), RPR qualitative (nonreactive), C-reactive protein (CRP, < 6 mg/L, normal), and erythrocyte sedimentation rate (ESR, 5 mm/h with normal at 0–15), though the latter two were sampled on day 20 post-oral steroids. A whole abdomen ultrasound done day 39 post-laser revealed splenomegaly and a gall bladder polyp versus encrusted stone. Additional tests including serum HLA-B27 testing and vitreous aspirate serology and immunologic testing were contemplated but not done due to unavailability in our center, as well as the limitations in the resources of the patient’s family.

As of this writing, at almost 12 weeks post-laser, uncorrected vision is 20/20, with the persistent anterior chamber reaction. On indirect ophthalmoscopy, sheet-like membranes are seen arising from the mid-periphery, obscuring the rest of the anterior/peripheral fundus.

The chronology of key events is enumerated in Table 1.

**Table 1:** Chronology of major events

Week 1	Day 1 post-laser
	• *Hand motion, light perception in all quadrants vision*
	• + 3 brown cells, + 2 flare in anterior chamber; dense vitreous haze on dilated indirect ophthalmoscopy
	• IOP 20 mmHg treated eye, 10 mmHg RRD eye
	• Topical polymyxin + neomycin + dexamethasone shifted to prednisolone acetate 10 mg/mL q2 hours, atropine sulfate 1% 3×/day, and oral acetazolamide 125 mg 2×/day
	Day 2
	• B-scan: low amplitude, mobile echoes in the mid to posterior vitreous, and prominent choroid (Fig. 1)
	Day 4
	• 1-mm blood-tinged hypopyon (Fig. 2)
	• IOP of 10 mmHg
	• Topical prednisolone acetate increased to one drop hourly, topical moxifloxacin 5 mg/mL 6 drops q3 hours started
	• Subconjunctival triamcinolone acetonide (10 mg)
	Day 6
	• Patient admitted to hospital
	Day 8
	• Oral prednisone 60 mg daily
Week 2	• Cells and flare decreased to between + 1 and + 2,
	• Hypopyon resolved day 13 (Fig. 3)
Week 3	• Pupillary synechiae, iris swelling 6–12-o’clock areas; dense, mobile, gray-brown retrolental sheets/membranes (Fig. 4)
	• **B-scan**: dense vitreous cells greatest towards posterior vitreous; mobile mid-amplitude echoes in nasal wall suspicious for exudative retinal detachment (Fig. 5)
	• **Ultrasound biomicroscopy**: ciliary body cyst-like lesion and swelling, echoes in the space between the iris and lens 4–10 o’clock (Fig. 6)
	• Oral immunosuppressive therapy, pars plana vitrectomy contemplated ➔ deferred due to stable condition, fear of pushing eye into phthisis
Week 4	• **B-scan**: same features but decreased amplitude of echoes
	• *20/200 vision* with and without pinhole on day 27
	• *20/70 correcting to 20/20 on pinhole* vision on day 36 (Fig. 7)
	• Fundus details first seen on indirect ophthalmoscopy day 30
Week 6	• Oral prednisone gradually adjusted to 40–50 mg/day
Week 8	• *20/20 uncorrected vision* on day 59 with persistence of + 1 to 2 anterior chamber cells and flare, retrolental membrane (Fig. 11)
	• **Macular OCT**: generalized macular thickening > 99th percentile, preserved foveal anatomy, very visible posterior hyaloid face detached above the foveal depression (Fig. 8)
	• **Fluorescein angiography**: normal dye transit, focal non-specific parafoveal staining, no evidence of vasculitis, phlebitis, delayed filling, gross macular edema nor ischemia (Figs. 9 and 10)
	• **B-scan**: near-total resolution of previously observed lesions
Week 11	• IOP ~ 25 treated eye, 30–35 RRD eye
	• Laser iridotomy and/or phacoemulsification contemplated ➔ deferred until resolution of inflammation
	• Timolol maleate 5 mg/mL 2×/day started
	• IOP decreased over following 2 days: 5–9 RRD eye, 17–19 treated eye (Figs. 11 and 12)

### Discussion

Non-traumatic detachment in one eye is associated with 10% risk of rhegmatogenous retinal detachment (RRD) in the fellow eye [1]. Should the initial retinal detachment be rhegmatogenous in nature, as high as 100 times greater risk of RRD in the fellow eye has been observed [2]. As a seeing, clinically silent eye, conservative management in this patient’s treated eye via periodic funduscopy was a valid option. This was predicated, however, on his ability to report for examination regularly and immediately so once with visual symptoms. The patient’s indigency, place of residence half a day away by public transportation from our center, along with such a degree of fellow eye retinal detachment risk were for us sufficient to advocate for aggressive intervention.

Widely accepted guidelines advocating prophylactic laser exist for focal lesions but not diffuse peripheral laser for any particular lesion. The chosen intervention, encircling laser retinopexy/cerclage, is done multiple times a week in our center. While there is skepticism of its efficacy, numerous studies have demonstrated greater than 50% risk reduction of later detachment both in the clinical/office/slit lamp and intraoperative (retinal detachment surgery) setting. Done in the hopes of “fencing out” future areas of detachment, and/or strengthening peripheral chorioretinal adhesion, no vision-threatening outcome following this specific intervention (aside from “failure,” i.e., later detachment) appears to have been reported in the literature [1–4].

A severe and protracted panuveitis-like reaction immediately followed this treatment, done to an eye with no documented previous interventions. No systemic comorbidities that may predispose to inflammation including diabetes were established. He is an able-bodied young adult male, a construction worker for whom our rheumatology service could not establish a definite rheumatologic disease entity to accompany any uveitic entity.

While the pre-laser peripheral vitreous condensations may have represented pre-existing intermediate uveitis, no cells, flare nor keratic precipitates in the anterior segment of the either eye, and the fundus of the contralateral eye were observed. An alternate hypothesis is that these condensations may have been organizing proliferative vitreoretinopathy from traumatic breaks we failed to visualize. With the history of head trauma, force threatening chorioretinal integrity was probably imparted to the fellow eye as well. In any event, this clinical sign was not felt to signify inflammation as in fact, it was initially signed out as benign vitreous condensations. Save for the established, self-limiting complications as in panretinal photocoagulation, no severe “blinding” complication was anticipated [1]. In retrospect, this sign may thus have been a cause to favor conservative management or, at least, more measured laser therapy.

Extensive retinal photocoagulation has been known to result in severe structural damage to the choroid and retina. Among these are retinal or Bruch’s membrane ruptures, as well as later retinal hole formation. Along with hemorrhages at all levels, blood-retinal or blood-aqueous barrier (BRB or BAB) breakdown is also known to occur [5–7]. Reports of similarly pronounced, acute post-laser inflammation have been documented in three cases where panretinal photocoagulation (PRP) was done for diabetic patients bearing varied clinical characteristics. All authors termed the phenomenon as “hypopyon uveitis,” with one case involving PRP following scleral buckling and vitrectomy (with 20/200 pre-laser vision) and the two others involving patients with opposite levels of glycemic control. The patient with poor glycemic control underwent laser indirect ophthalmoscopy with 450 spots, while the patient with good glycemic control as well as a history of acute anterior uveitis 1 and 3 years prior underwent slit lamp laser with 3300 spots delivered over two sittings, 1 week apart. Respectively, power settings were 190 and 300 mW, and duration settings were 200 and 30 ms. Symptoms were reported on day 1 and day 18 post-laser respectively and BCVA in both cases never worsened beyond 20/40. Unlike our case, purely topical anti-inflammatory therapy was sufficient to afford resolution of inflammatory signs and improvement in vision in less than 1 month for both cases [8, 9]. All researchers pointed to BAB breakdown as the probable mechanism for the events, which is highly tenable given its documentation in the acute post-laser period by earlier studies, as well as the setting of diseased vascular permeability barriers of diabetes [8–10].

Whether the pre-laser condensations represented a previous inflammatory episode, or vitreoretinopathy, this case may demonstrate how severe the reaction of a relatively young eye with either pathology (or even neither) could be to heavy laser work. Whatever its nature, such a finding may therefore call for more measured treatment in the form of multiple sessions and lower settings. The laser done was, arguably, comparable to a single-sitting PRP. Though with relatively weaker settings compared to (1) these previous reports of “hypopyon uveitis,” as well as (2) our usual PRP settings for diabetic retinopathy laser on this specific machine, anterior laser accomplishing near-confluent grade 3 burns may still have imparted comparatively greater energy to the eye [7–9]. No abnormal or unexpected performance nor post-laser reactions have been reported for the machine used.

Vitreous hemorrhage was probably a significant component of the pathology. In the absence of other systemic signs and symptoms of a bleeding disorder, managing services did not investigate this possibility further and did not obtain bleeding/clotting parameters. In any case, resulting/accompanying inflammation was so severe that anti-inflammatory therapy was our focus. Our hope was that as we were shielding the eye from concurrent inflammation, natural mechanisms might lead to resolution of any hemorrhage. Vitrectomy was contemplated to remove inflammatory debris, clear the visual axis, and afford visualization of the posterior pole. Theoretically, it is the sole direct intervention for a vitreous hemorrhage. Such intense inflammation with fluctuating intraocular pressures, however, bred fear of vitrectomy pushing the eye into phthisis. Hence, our strategy was steroid therapy and close observation (inpatient care), with systemic immunosuppressive agents on standby. For any surgery, we prefer an eye with minimum signs of inflammation, particularly anterior segment cells and flare, and no signs of exudative retinal detachment. Inpatient care allowed us to document improving visual acuity and B-scan picture, particularly over the critical first month. This permitted us to maintain our strategy, and with reestablished view of the posterior pole, we now reserve any surgery for a much later date.

Steroid therapy was increased incrementally as the severity of the inflammation was totally unexpected. A step-up was done on each day of follow-up in the first 8 days post-laser—switching from topical dexamethasone to prednisolone on day 1, increasing frequency of drops and supplementing with a subconjunctival triamcinolone depot on day 4, and, finally, starting oral prednisone on day 8. Unlike previous cases as well, multiple routes/modalities were employed. In retrospect, the earlier addition of oral steroids and even use of immunosuppressive agents may have been beneficial. Deliberations on the use of the latter and vitrectomy in the period day 15–25 were ultimately abandoned owing to the improving vision and ultrasound picture.

Despite the recovery of vision, the elements for numerous downstream complications are already at play. A mixed mechanism glaucoma and cataract due to persistent anterior segment inflammation and chronic steroid therapy may already have begun. Breaks and retinal detachment from any holes that may have resulted may arise, particularly given the patient’s young vitreous. Neovascular membranes and epiretinal membranes may also arise given the virtual injury to the posterior pole [6, 7]. We thus submit this case as a rare in vivo demonstration of the human eye’s response to heavy retinal photocoagulation. It would thus, at the very least, serve to demonstrate the need for caution and temperance in the conduct of any retinal photocoagulation, prudent timing of post-laser follow-ups, and, prompt and close inter-subspecialty collaboration following severe reactions to posterior segment laser.
